# Employing a latent variable framework to improve efficiency in composite endpoint analysis

**DOI:** 10.1177/0962280220970986

**Published:** 2020-11-24

**Authors:** Martina McMenamin, Jessica K Barrett, Anna Berglind, James MS Wason

**Affiliations:** 1 47959 MRC Biostatistics Unit, University of Cambridge, Cambridge, UK; 2 Late RIA, R&D BioPharmaceuticals AstraZeneca, Gothenburg, Sweden; 3 Institute of Health and Society, 5994 Newcastle University, Newcastle, UK

**Keywords:** Composite endpoint, latent variable model, responder analysis, systemic lupus erythematosus

## Abstract

Composite endpoints that combine multiple outcomes on different scales are common in clinical trials, particularly in chronic conditions. In many of these cases, patients will have to cross a predefined responder threshold in each of the outcomes to be classed as a responder overall. One instance of this occurs in systemic lupus erythematosus, where the responder endpoint combines two continuous, one ordinal and one binary measure. The overall binary responder endpoint is typically analysed using logistic regression, resulting in a substantial loss of information. We propose a latent variable model for the systemic lupus erythematosus endpoint, which assumes that the discrete outcomes are manifestations of latent continuous measures and can proceed to jointly model the components of the composite. We perform a simulation study and find that the method offers large efficiency gains over the standard analysis, the magnitude of which is highly dependent on the components driving response. Bias is introduced when joint normality assumptions are not satisfied, which we correct for using a bootstrap procedure. The method is applied to the Phase IIb MUSE trial in patients with moderate to severe systemic lupus erythematosus. We show that it estimates the treatment effect 2.5 times more precisely, offering a 60% reduction in required sample size.

## 1 Introduction

Composite endpoints combine a number of individual outcomes in order to determine the effectiveness or efficacy of a treatment for a given disease. A subset of these endpoints are composite responder endpoints in which patients are classed as ‘responders’ or ‘non-responders’ based on whether they cross predefined thresholds in the individual outcomes. These endpoints are common in autoimmune diseases such as systemic lupus erythematosus (SLE), lupus nephritis and sjögrens syndrome. Physicians and health authorities advocate these endpoints as they attempt to capture the effect in multiple dimensions of the disease. For instance, in SLE the composite is used to ensure that as improvement occurs in the SLE Disease Activity Index (SLEDAI), there is no simultaneous worsening in any other organ domains. In other diseases, such as rheumatoid arthritis and myositis, the composite endpoints capture improvements from both a clinical and patient perspective.

The composite of interest may be a combination of continuous and discrete outcomes which are typically collapsed in to a single binary responder index and analysed using a logistic regression model, termed the standard binary method. The work by Wason and Seaman^1^ showed that analysing in this way solves problems with multiplicity, however, at the expense of large losses in efficiency due to discarding information on how close each patient was to the responder threshold. For a composite containing a single continuous and binary endpoint they proposed the augmented binary method, a likelihood-based approach using the theory of factorisation models. They factorised the joint distribution and fit a univariate model to each component of the factorisation. This accounts for correlations between the outcomes by including one response as a covariate in the model for the other response. In the graphical modelling literature, this has been termed the ‘conditional Gaussian distribution’.^2,3^ The augmented binary method has been shown to reduce the required sample size in clinical trials by approximately 35% in a range of applications where the composite is formed of one continuous and one binary outcome.^1,4–6^ For composites made up of multiple continuous, ordinal and binary outcomes, we hypothesise that we may further increase efficiency due to the additional information in continuous and ordinal components. However, one limitation of these methods beyond the bivariate scenario is the range of possibilities for the factorisations, with no consensus on how this should be determined. In the case of an endpoint with four components, this amounts to 24 possible factorisations, each of which may result in different conclusions.^7,8^ To model complex, higher dimensional composite endpoints, we require a more general joint modelling framework.

To achieve this, we propose adopting a correlated Gaussian distribution for the components by assuming that the discrete outcomes are manifestations of underlying continuous variables, subject to some threshold specifications.^9,10^ This framework dates back to Pearson (1904)^11^ in relation to his generalised theory of alternative inheritance and has received much consideration in the literature since, although the terminology has been largely inconsistent. In the graphical modelling literature, they have been termed ‘conditional grouped continuous models (CGCMs)’^12^ and elsewhere have been referred to as ‘multivariate ordered probit models’,^13^ ‘correlated probit models’^14^ and ‘generalised multivariate probit models’.^15^ The general mixed-data model for mixed nominal, ordinal, and continuous data also reduces to a CGCM in the absence of nominal outcomes.^16^ The application of CGCMs for a mixture of continuous and binary outcomes has featured throughout the statistics literature.^17–19^ A CGCM has also been proposed in clinical trials to deal with the problem of multiple continuous and binary co-primary endpoints, where a treatment effect must be achieved in all outcomes to conclude it is successful overall.^20,21^ Extensions have allowed for modelling continuous and ordinal variables, with applications such as developmental toxicology and the joint modelling of hybrid traits in genetics.^22–27^ Other work has combined employing a latent Gaussian distribution for the response variables and introducing latent variables in the model; however, these ideas are most applicable in the longitudinal setting.^8,14,28–30^

The purpose of this work is to employ the CGCM framework to a different end. Rather than using the latent Gaussian distribution to make inference on multivariate outcomes, we will use it to model the multiple components within a composite, while still making inference on the one-dimensional composite endpoint. By employing the latent structure to collapse the multiple outcomes after the model is fitted, rather than before, we aim to greatly improve efficiency whilst still providing the overall treatment effect on the composite.

The paper proceeds as follows. In Section 2 we discuss SLE, the motivating example for the methods. In Section 3 we introduce the latent variable model for our application and discuss how we conduct estimation and inference for the composite endpoint problem. In Section 4 we introduce the comparison methods. In Section 5 we compare the behaviour of the latent variable model with the augmented binary and standard binary methods, including the case when the key assumptions are not satisfied. In Section 6 we apply the methods to the Phase IIb MUSE trial in patients with moderate to severe SLE. Finally, in Section 7 we discuss our findings and make recommendations for use.

## 2 Motivating example

Table 1 shows examples of composite endpoints combining multiple criteria to define response. Responders in fibromyalgia must respond in two continuous and one ordinal component; however, responders in trials for frailty or soft tissue infections must respond in a total of five continuous and discrete components. In what follows, we will focus specifically on SLE as a motivating example however, the methods introduced will be relevant to other diseases using endpoints with a similar structure.

**Table 1.** Examples of diseases that use complex composite endpoints combining multiple discrete and continuous measures to determine effectiveness of a treatment including criteria for response and how each component is measured.

Disease	Responder endpoint	Measured by
Fibromyalgia	• Achieved a 30% improvement in pain	Electronic diary
	• 30% improvement in functional status	Subscale of Fibromyalgia Impact Questionnaire (FIQ)
	• Improved, much improved, or very much improved	7-point Patient Global Impression of Change (PGIC) scale
Frailty	• BMI <18.5 kg/m2 OR >10% weight loss since last wave	Weight and height
	• One positive answer to exhaustion questions	CES-D questionnaire
	• Low grip strength (M < 31.12 kg, F < 17.60 kg)	E.g. Jamar hand dynamometer
	• Gait speed (M < 0.691 m/s, F < 0.619 m/s)	Distance/time
	• Low activity (M < 16.5 activity units, F < 13.5 activity units)	Activity units derived using intensity versus frequency
Necrotising soft	• Alive until day 28	Yes/No
tissue infections	• Day 14 debridements ≤ 3	Surface area
	• No amputation if debridement	Yes/No
	• Day 14 mSOFA score ≤ 1	mSOFA score – composite additively
	• Reduction of at least 3 score points in mSOFA score	combining scores in different systems mSOFA score – composite additively combining scores in different systems
Systemic lupus	• Change in SLEDAI ≤ −4	SLE Disease Activity Index
erythematosus	• Change in PGA < 0.3	Physicians Global Assessment
	• No Grade A or more than one	British Isles Lupus Assessment Group
	Grade B in BILAG	
	• Reduction in oral corticosteroids	Medical Notes

In the SLE endpoint, a continuous Physician’s Global Assessment (PGA) measure, a continuous SLEDAI measure and an ordinal British Isles Lupus Assessment Group (BILAG) measure are combined to form the SLE Responder Index (SRI).^31^ This is combined with a binary measure, which indicates tapering of the oral corticosteroids dose, to form the overall SLE responder endpoint of interest. The BILAG measure is a translational index which measures changing severity of clinical manifestations in nine organ systems. It has five levels for each parallel organ system, labelled Grade A–Grade E.^32^ Patients must meet the response criteria in all components in order to be classed as a responder overall. A figure denoting the structure of the SLE responder endpoint is shown in Appendix A of the supplemental material.

## 3 Methods

### 3.1 Model

The mean structure for the outcomes is shown in equation (1). The baseline measures *y*_10_ and *y*_20_ are included in the model for *Y*_1_ and *Y*_2_, respectively.

(1) $$\begin{array}{l} Y_{i1}=\alpha_{0}+\alpha_{1}T_{i}+\alpha_{2}y_{i10}+\varepsilon_{i1}\\ Y_{i2}=\beta_{0}+\beta_{1}T_{i}+\beta_{2}y_{i20}+\varepsilon_{i2}\\ Y_{i3}^{*}=\gamma_{1}T_{i}+\varepsilon_{i3}^{*}\\ Y_{i4}^{*}=\psi_{0}+\psi_{1}T_{i}+\varepsilon_{i4}^{*} \end{array}$$

The observed discrete variables are related to the latent continuous variables by partitioning the latent variable space, as shown in equation (2). The lower and upper thresholds for both discrete variables are set at $\tau_{03}=\tau_{04}=-\infty ,\tau_{53}=\tau_{24}=\infty$ and the binary cut-point is set at $\tau_{14}=0$ . The intercept term for the ordinal variable is set at $\gamma_{0}=0$ so that the cut-points $\tau_{13},\tau_{23},\tau_{33},\tau_{43}$ may be estimated. The intercept for the binary outcome *ψ*_0_ may be estimated as $\tau_{14}=0$ .

(2) $$Y_{i3}=\{\begin{array}{l} \text{Grade E}\text{if }\tau_{03}\leq Y_{i3}^{*}<\tau_{13},\\ \text{Grade D}\text{if }\tau_{13}\leq Y_{i3}^{*}<\tau_{23},\\ \text{Grade C}\text{if }\tau_{23}\leq Y_{i3}^{*}<\tau_{33},\\ \text{Grade B}\text{if }\tau_{33}\leq Y_{i3}^{*}<\tau_{43},\\ \text{Non-responder}\text{if }\tau_{43}\leq Y_{i3}^{*}<\tau_{53} \end{array}\;\;\;\;\;Y_{i4}=\{\begin{array}{l} 0,\text{if }\tau_{04}\leq Y_{i4}^{*}<\tau_{14},\\ 1,\text{if }\tau_{14}\leq Y_{i4}^{*}<\tau_{24} \end{array}$$

Following these assumptions, we can model the error terms in equation (1) as multivariate normal with zero mean and variance–covariance matrix Σ, as shown in (3). Note that the error variances for $\varepsilon_{3}^{*},\varepsilon_{4}^{*}$ are $\sigma_{3}=1$ and $\sigma_{4}=1$ , however this does not represent a constraint on the model but rather a rescaling required for identifiability.

(3) $$(\varepsilon_{i1}^{\;},\varepsilon_{i2}^{\;},\varepsilon_{i3}^{*},\varepsilon_{i4}^{*})∼N(0,\Sigma )\;\Sigma =\left(\begin{array}{cccc} \sigma_{1}^{2} & \rho_{12}\sigma_{1}\sigma_{2} & \rho_{13}\sigma_{1} & \rho_{14}\sigma_{1}\\ \rho_{12}\sigma_{1}\sigma_{2} & \sigma_{2}^{2} & \rho_{23}\sigma_{2} & \rho_{24}\sigma_{2}\\ \rho_{13}\sigma_{1} & \rho_{23}\sigma_{2} & 1 & \rho_{34}\\ \rho_{14}\sigma_{1} & \rho_{24}\sigma_{2} & \rho_{34} & 1 \end{array}\right)$$

The joint likelihood contribution for patient i with, for instance, $Y_{i3}=$ Grade C and $Y_{i4}=0$ , can be factorised as shown below

(4) $$l\left(\theta ;Y_{i}^{*}\right)=f(Y_{il},Y_{i2};\theta )\int_{\tau_{23}}^{\tau_{33}}\int_{-\infty }^{0}f(Y_{i3}^{*},Y_{i4}^{*}|Y_{i1}^{\;},Y_{i2}^{\;};\theta )\;\text{d}y_{4}^{*}\;\text{d}y_{3}^{*}$$

where θ is a vector which contains all model parameters. Note that it is possible to evaluate the joint likelihood contribution for patient i using $f(Y_{i1},Y_{i2},Y_{i3}^{*},Y_{i4}^{*};\theta )$ ; however, factorising as in equation (4) may reduce computational times, particularly in high-dimensional models. This formulation also allows us to express the observed likelihood as shown in equation (5).

(5) $$\begin{array}{l} l(\theta ;Y)=\prod_{i=1}^{N}\prod_{w=1}^{5}\prod_{k=1}^{2}f(Y_{i1},Y_{i2};\theta )\\ \;{\left[pr\left(Y_{i3}=w,Y_{i4}=k|Y_{i1}=y_{i1},Y_{i2}=y_{i2};\theta \right)\right]}^{I\{Y_{i3}=w,Y_{i4}=k\}} \end{array}$$

The joint probability of patients having discrete measurements $Y_{i3}=w$ and $Y_{i4}=k$ must be multiplied over the five ordinal levels and two binary levels resulting in 10 combinations of the probabilities to be calculated as shown in equation (6)

(6) $$\begin{array}{l} pr\left(Y_{i3}=w,Y_{i4}=k|Y_{i1}=Y_{i1},Y_{i2}=Y_{i2};\theta \right)\\ =\Phi_{2}\left(\tau_{w3}-\mu_{3|1,2},\tau_{k4}-\mu_{4|1,2};\Sigma_{3,4|1,2}\right)-\Phi_{2}\left(\tau_{(w-1)3}-\mu_{3|1,2},\tau_{k4}-\mu_{4|1,2};\Sigma_{3,4|1,2}\right)\\ \;-\Phi_{2}\left(\tau_{w3}-\mu_{3|1,2},\tau_{(k-1)4}-\mu_{4|1,2};\Sigma_{3,4|1,2}\right)+\Phi_{2}\left(\tau_{(w-1)3}-\mu_{3|1,2},\tau_{(k-1)4}-\mu_{4|1,2};\Sigma_{3,4|1,2}\right) \end{array}$$

where $\Phi_{2}$ is the bivariate standard normal distribution function and $\mu_{3|1,2},\mu_{4|1,2}$ and $\Sigma_{3,4|1,2}$ are derived using the rules of conditional multivariate normality, resulting in equation (7).

(7) $$\begin{array}{c} \mu_{3|1,2}=\mu_{3}+\frac{\left(\rho_{13}-\rho_{12}\rho_{23}\right)}{\sigma_{1}\left(1-\rho_{12}^{2}\right)}\left(Y_{i1}-\mu_{1}\right)+\frac{\left(\rho_{23}-\rho_{12}\rho_{13}\right)}{\sigma_{2}\left(1-\rho_{12}^{2}\right)}\left(Y_{i2}-\mu_{2}\right)\\ \mu_{4|1,2}=\mu_{4}+\frac{\left(\rho_{14}-\rho_{12}\rho_{24}\right)}{\sigma_{1}\left(1-\rho_{12}^{2}\right)}\left(Y_{i1}-\mu_{1}\right)+\frac{\left(\rho_{24}-\rho_{12}\rho_{14}\right)}{\sigma_{2}\left(1-\rho_{12}^{2}\right)}\left(Y_{i2}-\mu_{2}\right) \end{array}$$

$$\begin{array}{l} \Sigma_{3,4|1,2}=\left(\begin{array}{cc} 1-\frac{\rho_{13}^{2}-2\rho_{12}\rho_{13}\rho_{23}+\rho_{23}^{2}}{1-\rho_{12}^{2}} & \rho_{34}-\frac{\rho_{13}\rho_{14}-\rho_{12}\rho_{13}\rho_{24}-\rho_{12}\rho_{14}\rho_{23}+\rho_{23}\rho_{24}}{1-\rho_{12}^{2}}\\ \rho_{34}-\frac{\rho_{13}\rho_{14}-\rho_{12}\rho_{13}\rho_{24}-\rho_{12}\rho_{14}\rho_{23}+\rho_{23}\rho_{24}}{1-\rho_{12}^{2}} & 1-\frac{\rho_{14}^{2}-2\rho_{12}\rho_{14}\rho_{24}+\rho_{24}^{2}}{1-\rho_{12}^{2}} \end{array}\right) \end{array}$$

We discuss the intuition for equation (6) in Appendix B of the supplemental material.

### 3.2 Estimation

As the variance parameters $\left(\sigma_{1},\sigma_{2}\right)$ are required to be greater than 0, we introduce parameters $\left(\omega_{1},\omega_{2}\right)$ such that $\sigma_{1}=\exp(\omega_{1})$ and $\sigma_{2}=\exp(\omega_{2})$ . This transformation ensures that the variance is above 0 whilst allowing the estimated parameters to take any real value. We must also ensure that the correlation parameters $\left(\rho_{12},\rho_{13},\rho_{14},\rho_{23},\rho_{24},\rho_{34}\right)$ are estimated within (−1,1) by introducing $\left(\omega_{12},\omega_{13},\omega_{14},\omega_{23},\omega_{24},\omega_{34}\right)$ , where

$$\begin{array}{l} \rho_{12}=2e\mathrm{xpit}(\omega_{12})-1,\rho_{13}=2e\mathrm{xpit}(\omega_{13})-1,\rho_{14}=2e\mathrm{xpit}(\omega_{14})-1,\\ \rho_{23}=2e\mathrm{xpit}(\omega_{23})-1,\rho_{24}=2e\mathrm{xpit}(\omega_{24})-1,\rho_{34}=2e\mathrm{xpit}(\omega_{34})-1 \end{array}$$

We fit the model in R by coding the likelihood function and probability of response. The bivariate distribution functions in equation (6) are estimated using ‘pmvnorm’, applying the method of Genz.^33^ The likelihood maximisation is conducted using a quasi-Newton method based on port routines and can be implemented using the ‘nlminb’ function in the ‘optimx’ package. This is the best performing method in this setting in terms of accuracy and convergence rate however, it is also the slowest. Note that model parameters for these types of models may also be estimated using weighted least squares in the ‘lavaan’ package in R however, it allows for less flexibility in fitting the model.^34^ We use the ‘hessian’ function in the ‘numDeriv’ package to calculate the Hessian matrix using Richardson extrapolation^35^ and obtain the covariance matrix of the model parameters by inverting the Hessian. We ensure finite sample positive definiteness through solving a constrained optimisation problem,^36^ where the nearest correlation matrix projection is used to compute the nearest correlation matrix. We achieve this using the ‘near PD’ function, which implements the algorithm of Higham.^37^

### 3.3 Inference

We wish to make inference on the probability of response. Let *S_i_* be an indicator for patient i denoting whether or not they achieved response defined by *S_i_* = 1 if $Y_{i1}^{\;}\leq \eta_{1},Y_{i2}^{\;}\leq \eta_{2},Y_{i3}^{*}\leq \eta_{3},Y_{i4}^{*}\leq \eta_{4}$ . Therefore

(8) $$P(S=1|T,y_{10},y_{20})=\int_{-\infty }^{\eta_{1}}\int_{-\infty }^{\eta_{2}}\int_{-\infty }^{\eta_{3}}\int_{-\infty }^{\eta_{4}}f_{Y^{*}}(Y^{*};T,y_{10},y_{20})\;\text{d}y_{4}^{*}\;\text{d}y_{3}^{*}\;\text{d}y_{2}\;\text{d}y_{1}$$

where $f_{Y^{*}}(Y^{*};.)$ is the multivariate normal density function for the observed and latent continuous measures. We obtain the integrand in equation (8) by using the fitted values of the parameters in the conditional mean and conditional covariance matrix in equation (7). Parameter estimates from these methods are maximum likelihood estimates and so we avail of asymptotic maximum likelihood theory. The integral in equation (8) is evaluated using the ‘R2Cuba’ package to obtain estimates for each patient, assuming they were treated ${\tilde{p}}_{i1}$ and not treated ${\tilde{p}}_{i0}$ . The odds ratio treatment effect is then defined as shown in equation (9).

(9) $$\tilde{\delta }=\frac{\left(\frac{\sum_{i=1}^{N}{\tilde{p}}_{i1}}{N-\sum_{i=1}^{N}{\tilde{p}}_{i1}}\right)}{\left(\frac{\sum_{i=1}^{N}{\tilde{p}}_{i0}}{N-\sum_{i=1}^{N}{\tilde{p}}_{i0}}\right)}$$

Note that we can easily define a risk difference or risk ratio using these quantities but in what follows we consider $\tilde{\delta }$ to be the effect of interest. The standard error estimates are obtained using the delta method. This requires the covariance matrix of the maximum likelihood estimates Cov( $\hat{\theta }$ ) and $\prime \prime \tilde{\delta }$ , the vector of partial derivatives of $\tilde{\delta }$ with respect to each of the parameter estimates. The variance of $\tilde{\delta }$ is obtained as shown in equation (10).

(10) $$\mathrm{Var}(\tilde{\delta })=(\prime \prime \tilde{\delta })TCov(\hat{\theta })(\prime \prime \tilde{\delta })$$

Another important consideration for the model is how to assess goodness-of-fit. We propose an extension to an existing method for application in this case, which is detailed in Appendix C in the supplemental material.

## 4 Comparison methods

### 4.1 Standard binary method

The standard binary method is a logistic regression on the overall responder index, as shown in equation (11)

(11) $$\mathrm{logit}(Pr(S_{i}=1|T_{i},y_{i10})=\alpha_{0}+\alpha_{1}T_{i}+\alpha_{2}y_{i10}+\alpha_{3}y_{i20}$$

The maximum likelihood estimates and the covariance matrix can be used directly to estimate the odds ratio and standard error.

### 4.2 Augmented binary method

The augmented binary method is a joint modelling approach which retains the information from one continuous component and combines the remaining outcomes to form a binary response outcome. The model is shown below where the baseline measures for $Y_{i1}$ and $Y_{i2}$ are included for comparison, as they are accounted for in the mean structure of the latent variable method. As one time point is modeled, we can use a linear model for $Y_{i1}$ as shown in equation (12)

(12) $$Y_{i1}=\alpha_{0}+\alpha_{1}T_{i}+\alpha_{2}y_{i10}+\alpha_{3}y_{i20}+\varepsilon_{i}$$

where ε∼N(0,σ) . In this case, the failure time binary indicator will contain information from the remaining three components. *F_i_* is set equal to 0 if $Y_{i2}\leq \eta_{2},Y_{i3}$ is Grade B–E and $Y_{i4}=0$ , otherwise the patient is labelled a non-responder in these components and $F_{i1}=1$ . *F_i_* is modelled using the logistic regression model in equation (13). Note that as this method retains the additional information contained in only one of the continuous measures, the most informative continuous outcome should be chosen.

(13) $$\mathrm{logit}(Pr(F_{i}=1|T_{i},y_{i10},y_{i20})=\beta_{0}+\beta_{1}T_{i}+\beta_{2}y_{i10}+\beta_{3}y_{i20}$$

Maximum likelihood estimates for the parameters are obtained from fitting models (12) and (13). As in the latent variable method, equation (14) is used to obtain probability of response estimates for each patient, assuming they were treated ${\tilde{p}}_{i1}$ and not treated ${\tilde{p}}_{i1}$ .

(14) $$P(Y_{1}\leq \eta_{1},F_{1}=0|T,y_{10},y_{20})\;=\int_{-\infty }^{\eta_{1}}P(F_{1}=0|T,y_{10},y_{20})f_{Y_{1}}(y_{1};T,y_{10},y_{20})\;\text{d}y_{1}$$

As before, these quantities are used to define an odds ratio, risk ratio or risk difference.

## 5 Simulation study

### 5.1 Data generating model

Initially, we investigate the properties of the methods when the assumptions of the latent variable model are satisfied. The parameter values in the ‘baseline’ scenario are chosen to simulate the settings where composite endpoints are typically recommended for use. Namely, that all components contribute to classifying responders and non-responders and that components are coherent but not perfectly correlated. The parameter values have been informed by the MUSE trial dataset, in particular the correlation structure. The response probability in the control arm is 0.28 and in the treatment arm is 0.38, resulting in an odds ratio approximately equal to 1.60. The parameter values selected for the model in equation (1) are shown in Appendix D of the supplemental material. From this baseline case, we vary parameters to determine how the methods behave under various scenarios of interest. In particular, we vary the treatment effect, the responder threshold and the drivers of response. The parameter values for these data generating models are also included in Appendix D.

### 5.2 Performance criteria

The methods are evaluated against a range of performance criteria. The bias of the methods is calculated using $\frac{1}{n_{\mathrm{sim}}}\sum_{j=1}^{n_{\mathrm{sim}}}{\hat{\delta }}_{j}-\delta$ , where ${\hat{\delta }}_{j}$ is the estimated treatment effect in repetition j, *δ* is the true treatment effect and *n_sim_* is the number of simulated datasets. We assess the coverage of the methods using $\frac{1}{n_{\mathrm{sim}}}\sum_{j=1}^{n_{\mathrm{sim}}}1({\hat{\delta }}_{\mathrm{low},j}\leq \delta \leq {\hat{\delta }}_{\mathrm{upp},j})$ where ${\hat{\delta }}_{\mathrm{low},j}$ and ${\hat{\delta }}_{\mathrm{upp},j}$ are the estimated lower and upper confidence interval limits in repetition j. The power is evaluated using $\frac{1}{n_{\mathrm{sim}}}\sum_{j=1}^{n_{\mathrm{sim}}}1(p_{j}<\alpha )$ , where *p_j_* is the *p*-value returned by the *j*th repetition and *α* is the nominal significance level. We are also interested in the relative precision of the methods, which is obtained using $\frac{\hat{\mathrm{Var}}{\left({\hat{\delta }}_{j}\right)}_{B}}{\hat{\mathrm{Var}}{\left({\hat{\delta }}_{j}\right)}_{A}}$ , where $\hat{\mathrm{Var}}{\left({\hat{\delta }}_{j}\right)}_{A}$ and $\hat{\mathrm{Var}}{\left({\hat{\delta }}_{j}\right)}_{B}$ are the estimated variances of the estimated treatment effect in repetition j for method A and method B, respectively.

Another useful measure for evaluating performance is the bias-corrected coverage.^38^ It is obtained using $\frac{1}{n_{\mathrm{sim}}}\sum_{j=1}^{n_{\mathrm{sim}}}1({\hat{\delta }}_{\mathrm{low},j}\leq \bar{\delta }\leq {\hat{\delta }}_{\mathrm{upp},j})$ , where $\bar{\delta }$ is the mean ${\hat{\delta }}_{j}$ . By assessing coverage using $\bar{\delta }$ as the true treatment effect, we can determine whether poor coverage is due to bias in the treatment effect estimate or some other cause. If coverage is not nominal but bias-corrected coverage is nominal then we can attribute poor coverage to bias and attempt to correct this.^38^

## 5.3 Results

### 5.3.1 Varying treatment effect

**Figure 1.**
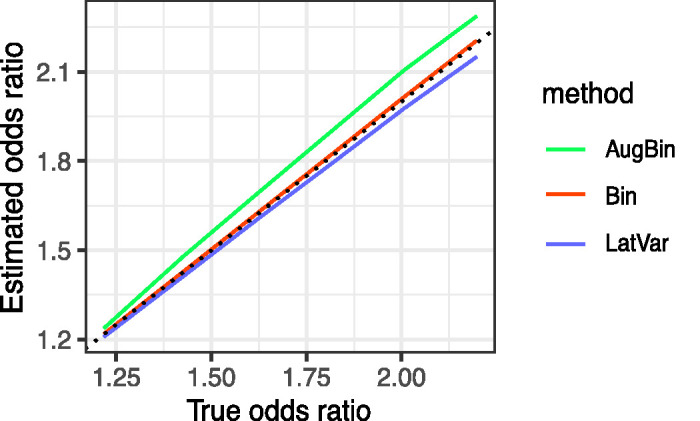
Bias reported from the latent variable method, augmented binary method and standard binary method when *n_sim_* = 5000, total sample size *n* = 300 for true odds ratio between 1.2 and 2.2. The composite endpoint of interest contains four components: two continuous, one ordinal, one binary and treatment effects are present in all four components.

Figure 1 shows the bias estimates for each method. The latent variable method is unbiased for smaller treatment effects but a small bias towards the null is introduced as the treatment effect increases. The augmented binary method is biased away from the null in this setting and the bias increases as the treatment effect increases. The standard binary method is unbiased, as we would expect for a logistic regression in a large sample.

The latent variable method has nominal coverage for smaller treatment effects; however, the coverage probability decreases as the treatment effect increases. The augmented binary method has coverage of approximately 0.91, which also decreases when the treatment effect increases. We find that the binary method has approximately nominal coverage.

**Figure 2.**
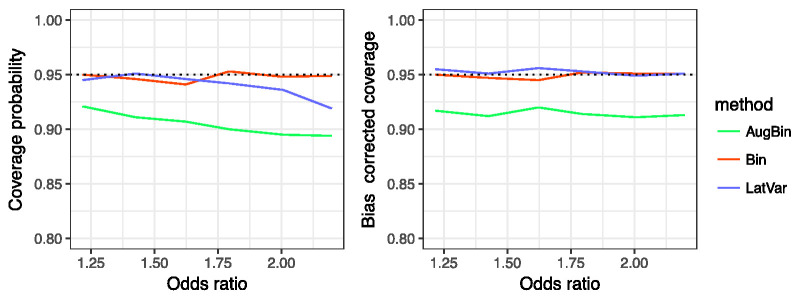
Coverage probability (left) and bias-corrected coverage probability (right) reported from the latent variable method, augmented binary method and standard binary method for *n_sim_* = 5000, and total sample size *n* = 300 for true odds ratio between 1.2 and 2.2. The composite endpoint of interest contains four components: two continuous, one ordinal, one binary and treatment effects are present in all four components.

Figure 2 shows both the coverage and bias-corrected coverage for the methods. The bias-corrected coverage of the latent variable method is 0.95, which indicates that any under-coverage is due to the bias present for larger treatment effect estimates. The augmented binary method shows small improvements in bias-corrected coverage, indicating that under-coverage is present in this method due to reasons other than bias. This may be due to model misspecification.

**Figure 3.**
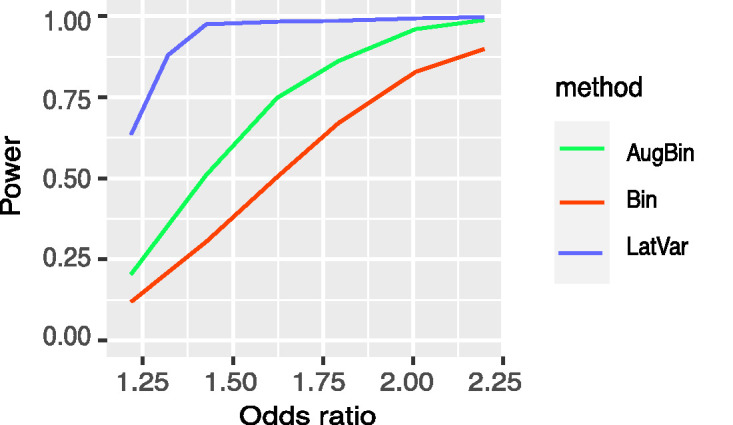
Statistical power reported from the latent variable method, augmented binary method and standard binary method for *n_sim_* = 5000, and total sample size *n* = 300 for true odds ratio between 1.2 and 2.2. The composite endpoint of interest contains four components: two continuous, one ordinal, and one binary, and treatment effects are present in all four components.

The power of the methods is shown in Figure 3. The performance of the binary and augmented binary methods is as we would expect based on previous findings.^1,4^ The latent variable method offers much higher power. In this setting it has close to 100% power for odds ratios larger than 1.6, an effect that is plausible to observe in a trial. The mean squared error (MSE) for the standard and augmented binary methods is approximately 6.5 times that of the latent variable method. The MSE plot is shown in Appendix E of the supplemental material.

### 5.3.2 Varying η_1_

To understand more about the precision performance of the augmented binary method in particular, we vary the responder threshold *η*_1_ to change the proportion of responders in that outcome. We find that the precision gains from the augmented binary method diminish as the threshold increases. This is intuitive, as gains in efficiency fall as the continuous component becomes less responsible for driving response. It is interesting to note that all precision gains are lost for any thresholds above −4. Therefore, even when 20% of patients are non-responders, all efficiency gains are lost. The percentage of responders needed to improve efficiency using the augmented binary method will of course depend on the correlation structure employed. Due to the additional information in the other components, the latent variable method is still five times as precise as the other methods. The results are shown in Appendix E.

### 5.3.3 Components contributing to response

**Figure 4.**
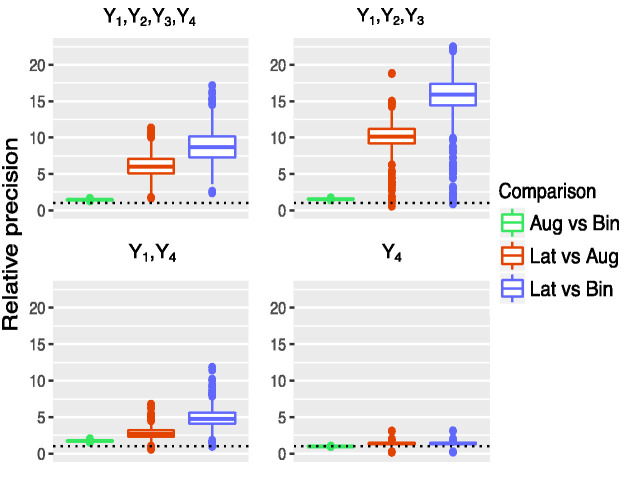
Estimated relative precision gains from augmented binary versus standard binary method, latent variable versus augmented binary method and latent variable versus standard binary method when different combinations of components are driving response. Response driven by ( $Y_{1},Y_{2},Y_{3},Y_{4}$ ), ( $Y_{1},Y_{2},Y_{3}$ ), (*Y*_1_, *Y*_4_) and (*Y*_4_) where *Y*_1_ and *Y*_2_ are continuous, *Y*_3_ is ordinal, *Y*_4_ is binary for *n_sim_* = 5000 and total sample size *n* = 300. The composite endpoint of interest contains four components: two continuous, one ordinal, and one binary, and treatment effects are present in all four components.

Figure 4 shows boxplots of the relative precision of the methods for four different response combinations, namely when response is driven by $\left(Y_{1},Y_{2},Y_{3},Y_{4}),(Y_{1},Y_{2},Y_{3}),(Y_{1},Y_{4}\right)$ and $\left(Y_{4}\right)$ , where *Y*_1_ and *Y*_2_ are observed as continuous variables, *Y*_3_ is ordinal and *Y*_4_ is binary.

When all four components contribute to response, the latent variable method outperforms the other methods, offering large precision gains. The variability in the magnitude of these gains is large, with the median result showing that the latent variable method reports the treatment effect eight times more precisely than the binary method and six times more precisely than the augmented binary method. If response is driven by $\left(Y_{1},Y_{2},Y_{3}\right)$ then the relative median gains for the latent variable method are larger; however, note that in less than 2% of cases the treatment effect is reported equally or less precisely than from both of the other methods. The findings are similar when response is driven by $\left(Y_{1},Y_{4}\right)$ , however the median gains are much smaller. The treatment effect is reported five times more precisely from the latent variable method than the binary method in this setting. Note that as the augmented binary method models the relevant components, it still performs well and again better than the latent variable method in a very small number of cases. When binary *Y*_4_ determines response, the augmented binary method offers no improvement in precision whereas the latent variable method is approximately 1.5 times more precise.

### 5.3.4 Sensitivity analysis

The key assumptions in this model are joint normality of the four components and that discrete variables can be modelled as latent continuous variables. Although it is not possible to test these assumptions in real data, we can investigate how robust the latent variable method is to deviations from these conditions. We do this by drawing from the multivariate skew-normal distribution with different degrees of skew in each of the components. The first scenario investigated considers when all four components are skewed. Scenarios 2–3 consider different magnitudes of skew in the latent continuous components only. Scenario 4 considers skew in the latent continuous components only for the case when there is no treatment effect. The results are shown in Appendix F of the supplemental material.

In summary, scenarios 1–3 have increased bias resulting in under-coverage as the bias-corrected coverage is close to nominal for all scenarios. The coverage of the latent variable method is nominal in the null case. This introduction of potentially large biases in the treatment effect estimate may be problematic for practice; however, it will result in a more conservative treatment effect estimate. The latent variable method still offers large power gains over the other methods and the MSE is the smallest for the latent variable method across all scenarios investigated. The latent variable method estimates the probability of response in the control arm well, however it underestimates the probability of response in the treatment arm. The magnitude of this underestimation is unaffected by the degree of skew or whether the skew is present in the observed continuous components. The relative precision of the methods are consistent with our previous findings indicating that the violation of joint normality only affects the bias and not the variance. The augmented binary and standard binary methods behave similarly to when the joint normality assumptions are satisfied, which is expected given that the assumptions of those models are potentially violated in both contexts.

## 6 Case study

### 6.1 Data structure

The real data underpinning this motivation comes from the MUSE study.^39^ It was a Phase IIb, randomised, double-blind, placebo-controlled study investigating the efficacy and safety of anifrolumab in adults with moderate to severe SLE. Patients (*n* = 305) were randomised to receive anifrolumab (300 mg or 1000 mg) or placebo, in addition to standard therapy every four weeks for 48 weeks. The primary end point was the percentage of patients achieving an SRI response at week 24 with sustained reduction of oral corticosteroids (<10 mg/day and less than or equal to the dose at week 1 from week 12 through 24). Due to data sharing policy, we conduct the analysis for a subset of the patients, *n* = 278 rather than *n* = 305 reported in the paper, so the results will differ from the original paper. Furthermore, only the anifrolumab 300 mg arm (*n* = 95) and the placebo arm (*n* = 87) will be used to illustrate the methods.

The simulation results have suggested that the structure of the data is important for how the methods will perform; in particular, the magnitude of the precision gains depends highly on which components drive response. In the case of the MUSE study, the components responsible for driving response are the continuous SLEDAI measurement and the binary taper measure. The structure of the data is further explored in Appendix G of the supplemental material.

## 6.2 Results

The probability of response in the placebo arm is estimated as 0.199 by the latent variable method, 0.211 by the augmented binary method and 0.224 by the standard binary method. A much larger discrepancy between the methods is shown in the treatment arm, where the probability of response is estimated at 0.311, 0.324 and 0.382 in the latent variable, augmented binary and standard binary methods, respectively.

The log-odds treatment effect point estimates and confidence intervals for the MUSE trial are shown in Table 2. Both joint modelling methods estimate the treatment effect more precisely. Although there may be bias present in the point estimates for the joint modelling methods, the confidence intervals entirely overlap with that of the binary method. All three methods indicate that anifrolumab 300 mg performs better than placebo, as in the original findings. The latent variable model fits the data well according to the modified Pearson residuals (see Appendix G, supplemental material).

**Table 2.** Log-odds treatment effect estimates and 95% confidence intervals from the latent variable method, augmented binary method and standard binary method in the Phase IIb MUSE trial and the bootstrap sample when *n* = 182 and *n*_boot_ = 1000

Method	Log-odds treatment effect	
	MUSE trial estimate	Bootstrap estimate
Latent variable	0.641 (0.217, 1.072)	0.682 (0.275, 1.137)
Augmented binary	0.580 (0.139, 1.021)	0.608 (0.096, 1.111)
Binary	0.763 (0.078, 1.449)	0.809 (0.112, 1.561)

The simulation results indicated that the latent variable method may report the treatment effect with bias when the effect is large and when the assumption of joint normality is not satisfied. Although the observed continuous outcomes are normally distributed, we were unable to assess joint normality due to the discrete components. As the problems with performance are bias related, we suggest implementing a bootstrap procedure to correct for this. The concept is based on treating the observed sample as the population and sampling with replacement from this. An estimate of the bias is obtained using the difference between treatment effects in the assumed population and the mean effect from the bootstrap samples.^40^ A theoretical justification for this procedure specific to latent variable and structural equation modelling, along with examples, is provided in the literature.^41,42^

In this scenario *n* = 182 and *n*_boot_ = 1000, therefore the procedure is as follows:

1. Sample with replacement *n* = 182 patients from the MUSE trial.
2. Compute the treatment effect using the latent variable, augmented binary and standard binary methods.
3. Repeat steps 1 and 2 *n*_boot_ = 1000 times.
4. Estimate bias as the difference in MUSE trial effect and mean of the bootstrap treatment effects.

A 95% bootstrap confidence interval for the treatment effect estimate can be obtained by ordering the 1000 bootstrap estimates of the treatment effect and taking the 25th and 975th estimate. The point estimates and 95% confidence intervals from the MUSE trial and from the bootstrap re-sampling are shown in Table 2.

The log-odds point estimate from the latent variable method has been shifted away from the null by approximately 0.04. This is in agreement with the magnitude of bias suggested by the simulation results. The point estimate for the binary method has also been shifted substantially which is likely due to the large imprecision in the treatment effect reported by the binary method. The latent variable method reports the treatment effect 2.5 times more precisely than the standard binary method in this setting, whilst the augmented binary method is 2.4 times more precise. We would have expected the methods to perform similarly as the augmented binary method models the only components driving response. This increase in precision from the latent variable method compared with the binary method amounts to a 60% reduction in required sample size.

## 7 Discussion

In this paper we addressed the issue of substantial losses of information when modelling complex composite endpoints. By partitioning latent variable outcome spaces, we could model the observed structure of the composite endpoint, which resulted in large gains in efficiency. Sensitivity analyses showed that a bias is introduced when the assumptions of joint normality were not satisfied; however, similar reductions in variance were observed. When applying the methods to the MUSE trial, we implemented a bootstrap procedure to correct for the presumed bias, as joint normality could not be assessed. The treatment effect was reported 2.5 times more precisely than that reported from the standard binary method.

Bias correction appears to perform well in the real data, where the assumptions cannot be tested. The point estimate is shifted by a magnitude that would have been expected from the simulation results and the estimate of the variance is similar to that obtained in the single trial dataset. Furthermore, the bootstrap confidence interval for the treatment effect is contained within that for the binary method, which offers further reassurance for application. However, more work could be done to investigate different structures and scenarios to ensure that the bias correction is always performing as expected. Ideally, we would investigate this further across a large number of datasets however this is too computationally intensive. To perform this on one replicate where *n*_boot_ = 1000 currently takes 7 h using 200 cores on a high performance computer (HPC). An alternative in this setting is to model the multivariate dependence between components using copulas.^43^ Proceeding in this way would allow us to relax the Gaussian assumption and instead join the multivariate distribution functions to their one-dimensional marginal distribution functions. Given that we have shown the latent variable model to be sensitive to these assumptions, exploring the application of copulas in the composite endpoint setting is an important area for future research.

The precision gains offered by the latent variable method offer justification for the additional complexity, however the magnitude of these gains are highly dependent on the components that drive response. In the baseline scenario where all components drive response, the latent variable method reported the effect 2.5 to 17.5 times more precisely than the standard binary method. However, in practice in SLE trials all four components have not been found to drive response. A review of two phase 3 trials (*n* = 2262) using the SRI-5 index found that the SRI-5 response rate at week 52 for all patients was 32.8%.^44^ Non-response due to a lack of SLEDAI improvement, concomitant medication non-compliance or dropout was 31, 16.5 and 19.1%, respectively. Non-response due to deterioration in BILAG or Physician’s Global Assessment after SLEDAI improvement, concomitant medication compliance and trial completion was 0.5%. This is in agreement with our findings from the MUSE trial data, which suggests that the precision gains in the baseline case are optimistic. The simulation results show that when one continuous and one binary components drive response, the latent variable method may be anywhere between 1 and 12 times as precise as the binary method and up to seven times as precise as the augmented binary method. In a very small number of cases (<2%), there are no efficiency gains from using the latent variable method in this scenario. However, the potential gains available in 98% of cases ensure that implementing the latent variable method is still very much a worthwhile endeavour for all stakeholders in a clinical trial.

In addition to SLE, we have identified other disease areas that have a similar complex composite structure, meaning the potential to improve efficiency extends well beyond SLE. However, it must be acknowledged that the exact structure of the endpoint may offer different magnitudes of bias and precision, and may require longer computational time. Furthermore, in conditions where longitudinal data is required to sufficiently capture disease activity, trials may include multiple follow-up times and the method will need to be extended to include latent variables in the mean structure to account for this. In terms of scalability to more complex endpoints, the computational time depends on many things, in particular the number of outcomes, the outcome scale and the number of levels in the ordinal variable. In our case, we find the number of ordinal levels to be the most influential factor. This is due to the fact that five levels in the ordinal variable leads to 10 probability calculations in equation (6); however three levels would require the computation of six joint probabilities. Consequently, the run time will be substantially increased if there are multiple ordinal outcomes and decreased if the discrete variables are binary. If the computational time for a particular endpoint is deemed to be too large, then we may reduce the complexity of the endpoint by collapsing the least informative components into a single binary variable. It must be acknowledged that as we have coded the method, the likelihood and probability of response code will have to be tailored specifically to each endpoint. The potential gains in efficiency justify this additional complexity.

Obtaining maximum likelihood estimates from latent variable models has been achieved in different ways throughout the literature. In this paper we use a quasi-Newton algorithm, however these and Newton type algorithms are not without their limitations, such as tending to be slow or intractable in higher dimensions.^14^ The EM algorithm has been proposed in this setting as it lends itself well to situations with unknown parameters such as the *τ*-thresholds, however, conditioning on these parameters as in equation (4) violates regularity conditions. Hence a Parameter-Expanded EM algorithm which transforms the latent variables and expands the parameter space may be more appropriate.^45^ For an implementation of this estimation method when identifying genetic factors for comorbid conditions, we refer the reader to Zhang et al.^27^ Implementing the method as we have done in this paper is computationally demanding however, we would not expect the Parameter Expanded EM algorithm to rectify this and may actually lead to increased computational time. More work is required to compare estimation methods for these latent variable models.

We have shown that the latent variable method is a powerful tool in composite endpoint analysis and should be considered as a primary analysis method in a trial using these endpoints. In order for implementation in the general case, where the composite contains any number of continuous and discrete outcomes, we have developed a web based Shiny application, as detailed below. Furthermore, in order for patients and investigators to benefit from the efficiency gains, our current work is focused on developing a method for sample size calculation using these models, along with software to implement this.^46^ Our future work involves extending the method to include count and time-to-event endpoints for more general application.

## Software

A Shiny application for implementing the method is available at https://martinamcm.shinyapps.io/augbin/. Documentation and example data are available at https://github.com/martinamcm/AugBin.

## Supplemental Material

sj-pdf-1-smm-10.1177_0962280220970986 - Supplemental material for Employing a latent variable framework to improve efficiency in composite endpoint analysis Supplemental material, sj-pdf-1-smm-10.1177_0962280220970986 for Employing a latent variable framework to improve efficiency in composite endpoint analysis by Martina McMenamin, Jessica K Barrett, Anna Berglind and James MS Wason in Statistical Methods in Medical Research

## Appendix

### Notation

$Y_{i}={\left(Y_{i1},Y_{i2},Y_{i3},Y_{i4}\right)}^{T}$: vector of observed outcomes for patient i∈N
$Y={\left(Y_{1},\ldots Y_{N}\right)}^{T}$: observed outcomes for all patients
$Y_{i1}\;\text{ and }\;Y_{i2}$: observed continuous SLEDAI and PGA measures
$Y_{i3}$: observed ordinal BILAG measure
$Y_{i4}$: observed binary oral corticosteroid tapering measure
$Y_{i3}^{*}$: Latent continuous BILAG measure
$Y_{i4}^{*}$: Latent continuous oral corticosteroid tapering measure
$Y_{i}^{*}={\left(Y_{i1}^{\;},Y_{i2}^{\;},Y_{i3}^{*},Y_{i4}^{*}\right)}^{T}$: vector of observed and latent continuous measures for patient i
$Y^{*}={\left(Y_{1}^{*},\ldots Y_{N}^{*}\right)}^{T}$: Vector of observed and latent continuous measures for all patients
*T_i_*: treatment indicator for patient i
$y_{i10}\;\text{ and }\;y_{i20}$: baseline measures for $Y_{i1}$ and $Y_{i2}$ , respectively
