# Supplementary material for: Employing a latent variable framework to improve efficiency in composite endpoint analysis
Source: Stat Methods Med Res. 2020 Nov 24;30(3):702–16. doi: 10.1177/0962280220970986 (PMC8172253; doi:10.1177/0962280220970986)
Supplement: sj-pdf-1-smm-10.1177_0962280220970986 - Supplemental material for Employing a latent variable framework to improve efficiency in composite endpoint analysis [file sj-pdf-1-smm-10.1177_0962280220970986.pdf]

Journal Title
XX(X):1–18
©The Author(s) 0000
Reprints and permission:
sagepub.co.uk/journalsPermissions.nav
DOI: 10.1177/ToBeAssigned
www.sagepub.com/
SAGE

Supplementary Material: Employing a latent variable framework to improve efficiency in composite endpoint analysis

Martina McMenamin[1], Jessica K. Barrett[1], Anna Berglind[2] and James M.S. Wason[1,3]

[1]MRC Biostatistics Unit, University of Cambridge, UK
[2] Late RIA, R&D BioPharmaceuticals AstraZeneca, Gothenburg, Sweden
[3] Institute of Health and Society, Newcastle University, Newcastle, UK

Corresponding author:
Martina McMenamin, MRC Biostatistics Unit, University of Cambridge, Cambridge Institute of Public Health, Cambridge Biomedical Campus, Cambridge CB2 0SR UK
Email: martina.mcmenamin@mrc-bsu.cam.ac.uk

Appendix A

The structure for the systemic lupus erythematosus (SLE) endpoint is shown in Figure 1. Note that the SLEDAI and BILAG measures are both composite measures comprised of 102 and 24 items respectively. These are combined with the PGA measure to form the binary SRI composite[1]. This is then combined with a binary indicator containing information on patients corticosteroid use, which forms the overall SLE responder endpoint of interest. The order in which these components are combined has no bearing on the analysis, as patients must respond in each component at a given time-point to be classed as a responder at that time-point.

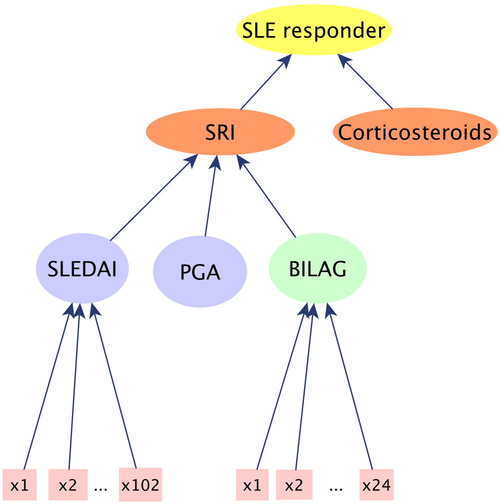

Figure 1. Structure of the composite endpoint used in trials of systemic lupus erythematosus. The continuous SLEDAI, continuous PGA and ordinal BILAG measures are dichotomised and combined to form the binary SRI indicator which is then combined with the binary taper variable to form the overall binary SLE responder index

Appendix B

The joint probability below expresses, for patient i with $Y_1 = y_{i1}$ and $Y_2 = y_{i2}$, the probability that they will have a Y_3 score w and a Y_4 score k.

$$\begin{aligned} &pr\left(Y_{i3} = w, Y_{i4} = k | Y_{i1} = Y_{i1}, y_{i2} = y_{i2}; \boldsymbol{\theta}\right) = \\ &\Phi_2\left(\tau_{w3} - \mu_{3|1,2}, \tau_{k4} - \mu_{4|1,2}; \Sigma_{3,4|1,2}\right) - \Phi_2\left(\tau_{(w-1)3} - \mu_{3|1,2}, \tau_{k4} - \mu_{4|1,2}; \Sigma_{3,4|1,2}\right) - \\ &\Phi_2\left(\tau_{w3} - \mu_{3|1,2}, \tau_{(k-1)4} - \mu_{4|1,2}; \Sigma_{3,4|1,2}\right) + \Phi_2\left(\tau_{(w-1)3} - \mu_{3|1,2}, \tau_{(k-1)4} - \mu_{4|1,2}; \Sigma_{3,4|1,2}\right) \end{aligned} \tag{B.1}$$

The intuition for the joint probability can be seen below in Figure 2, specifically for the SLE endpoint, where $w = 5$ and $k = 2$.

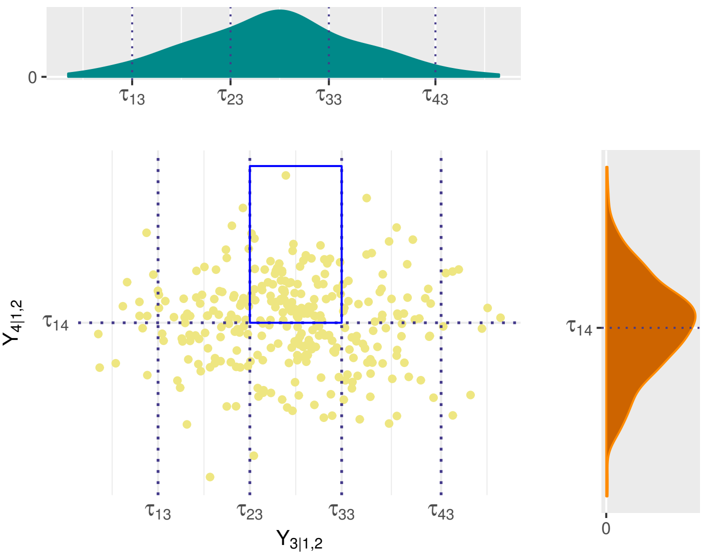

Figure 2. The figure shows the conditional outcome $Y_{3|1,2}$ on the x-axis and $Y_{4|1,2}$ on the y-axis with their corresponding underlying continuous densities and partitioning thresholds. The area where $w = 3$ and $k = 2$ is highlighted for illustration

The blue box indicates the region where $w = 3$ and $k = 2$. As $\tau_{03} = \tau_{04} = -\infty$ and $\tau_{53} = \tau_{24} = \infty$, the corresponding probability is shown in (B.2).

$$pr\left(Y_{i3}=3,Y_{i4}=2|Y_{i1}=y_{i1},Y_{i2}=y_{i2};\boldsymbol{\theta}\right)=$$
$$\Phi_2\left(\tau_{33}-\mu_{3|1,2},\infty-\mu_{4|1,2};\Sigma_{3,4|1,2}\right)-\Phi_2\left(\tau_{23}-\mu_{3|1,2},\infty-\mu_{4|1,2};\Sigma_{3,4|1,2}\right)-$$
$$\Phi_2\left(\tau_{33}-\mu_{3|1,2},\tau_{14}-\mu_{4|1,2};\Sigma_{3,4|1,2}\right)+\Phi_2\left(\tau_{23}-\mu_{3|1,2},\tau_{14}-\mu_{4|1,2};\Sigma_{3,4|1,2}\right) \tag{B.2}$$

Appendix C

One suggestion in the literature for assessing goodness-of-fit in latent variable models is introduced by[2] for the case when there is one continuous and one ordinal variable. This may be extended to allow for two continuous, one ordinal and one binary outcome for application in SLE, as shown below.
As before, let $\mathbf{Y}_i = (Y_{i1}, Y_{i2}, Y_{i3}, Y_{i4})'$ be the vector of observed responses for patient i. Then, partitioning the observed and latent continuous measures, we let $\mathbf{Y_{cts}} = (Y_1, Y_2)$ and $\mathbf{Y_{dis}} = (Y_3, Y_4)$. Then, $\hat{\Sigma}_{11} = \hat{Var}(\mathbf{Y_{cts}}), \hat{\Sigma}_{22} = \hat{Var}(\mathbf{Y_{dis}}), \hat{\Sigma}_{12} = \hat{\Sigma}_{21} = \hat{Cov}(\mathbf{Y_{cts}}, \mathbf{Y_{dis}})$.
The modified Pearson residuals, taking in to account the correlation between responses are shown below.

$$r_i^p = \hat{\Sigma}^{-\frac{1}{2}}(Y_i - \hat{\mu}_i) \tag{C.1}$$

where,

$$\hat{\boldsymbol{\mu}}_{\boldsymbol{i}} = (\hat{E}(Y_{i1}, Y_{i2} \mid X_{i1}, X_{i2}), \hat{E}(Y_{i3}, Y_{i4} \mid X_{i3}, X_{i4}))' \tag{C.2}$$

and

$$\hat{\Sigma} = \begin{pmatrix} \hat{\Sigma}_{11} & \hat{\Sigma}_{12} \\ \hat{\Sigma}_{21} & \hat{\Sigma}_{22} \end{pmatrix} \tag{C.3}$$

A Cholesky decomposition may be used to obtain $\hat{\Sigma}^{-\frac{1}{2}}$ in (C.1). The covariance between the vector of observed continuous and observed discrete responses is shown below.

$$\begin{aligned}
\Sigma_{12} =& E(\mathbf{Y_{cts}}\mathbf{Y_{dis}}) - E(\mathbf{Y_{cts}})E(\mathbf{Y_{dis}}) \\
=& E(\mathbf{Y_{cts}}E(\mathbf{Y_{dis}} \mid \mathbf{Y_{cts}})) - E(\mathbf{Y_{cts}})E(\mathbf{Y_{dis}}) \\
=& E(Y_1 Y_2 E(Y_3, Y_4 \mid Y_1, Y_2)) - E(\mathbf{Y_{cts}})E(\mathbf{Y_{dis}}) \\
=& \int_{y_1}\int_{y_2} y_1 y_2 \sum_{y_3}\sum_{y_4} y_3 y_4 P\left(Y_3 = w, Y_4 = k | Y_1 = y_1, Y_2 = y_2\right) \\
& f_{Y_1,Y_2}(y_1, y_2) dy_1 dy_2 - E(\mathbf{Y_{cts}})E(\mathbf{Y_{dis}})
\end{aligned}$$

Where,

$$P\left(Y_3 = w, Y_4 = k | Y_1 = y_1, Y_2 = y_2\right) =$$
$$\Phi\left(\tau_{w3} - \mu_{3|1,2}, \tau_{k4} - \mu_{4|1,2}; \Sigma_{3,4|1,2}\right) - \Phi\left(\tau_{(w-1)3} - \mu_{3|1,2}, \tau_{k4} - \mu_{4|1,2}; \Sigma_{3,4|1,2}\right) -$$
$$\Phi\left(\tau_{w3} - \mu_{3|1,2}, \tau_{(k-1)4} - \mu_{4|1,2}; \Sigma_{3,4|1,2}\right) + \Phi\left(\tau_{(w-1)3} - \mu_{3|1,2}, \tau_{(k-1)4} - \mu_{4|1,2}; \Sigma_{3,4|1,2}\right)$$

$$E(\mathbf{Y_{cts}}) = \int_{y_1}\int_{y_2} y_1 y_2 f_{Y_1,Y_2}(y_1, y_2) dy_1 dy_2$$
$$E(\mathbf{Y_{dis}}) = \sum_{y_3}\sum_{y_4} y_3 y_4 P\left(Y_3 = w, Y_4 = k\right)$$

and

$$P(Y_3 = w, Y_4 = k) =$$
$$\Phi(\tau_{w3} - \mu_3, \tau_{k4} - \mu_4; \rho_{3,4}) - \Phi(\tau_{(w-1)3} - \mu_3, \tau_{k4} - \mu_4; \rho_{3,4}) -$$
$$\Phi(\tau_{w3} - \mu_3, \tau_{(k-1)4} - \mu_4; \rho_{3,4}) + \Phi(\tau_{(w-1)3} - \mu_3, \tau_{(k-1)4} - \mu_4; \rho_{3,4})$$

The Pearson residual is based on the Pearson goodness-of-fit statistics

$$\chi_p^2 = \sum_{i=1}^{n} \chi_p^2(Y_i, \hat{\mu_i}) \tag{C.4}$$

with ith component

$$\chi_p^2(Y_i, \hat{\mu_i}) = (Y_i - \hat{\mu_i})'\hat{\Sigma}^{-1}(Y_i - \hat{\mu_i}) \tag{C.5}$$

The distribution of the residuals should follow a chi-squared distribution with p degrees of freedom. Comparing the residuals to the chi-squared value allows us to identify observations which the model does not fit well. If there are many observations unexplained by the model then it could indicate a poor choice of model. This may be due to the covariance structure $\hat{\Sigma}$ and its assumed distribution. The model may be refitted with various covariance structures and to obtain a model which is found to satisfactorily explain the observed data. If this is not achieved then joint normality of the error terms may be an unreasonable assumption indicating that the latent variable model may not be appropriate. It is possible to fit latent variable models which assume a different multivariate distribution for the error terms, however this is not considered here.

Appendix D

Table 1 shows the parameter values used to investigate the various scenarios considered in the paper. Only the parameters mentioned in each scenario are varied, the remaining parameters assume values assigned in the baseline case. Table 2 shows the parameter values for the data generating model in the baseline simulation scenario. These were chosen based on parameter values present in the MUSE trial.

Table 1. Parameter values for the simulated scenarios which investigate the effect of varying responder threshold η_1, changing the components driving response and differing treatment effects on the performance of the latent variable, augmented binary and standard binary methods for the systemic lupus erythematosus composite endpoint

Scenario	Parameters	Investigates
$\eta_1 = -2$	$\eta_1 = -2$	100% of patients respond in Y_1
$\eta_1 = -3$	$\eta_1 = -3$	96% of patients respond in Y_1
$\eta_1 = -4$	$\eta_1 = -4$	82% of patients respond in Y_1
$\eta_1 = -5$	$\eta_1 = -5$	52% of patients respond in Y_1
$\eta_1 = -6$	$\eta_1 = -6$	20% patients respond in Y_1
Y_1, Y_4	$\eta_1 = -5, \eta_2 = 2, \eta_3 = 2$	Continuous and binary variable driving response
Y_4	$\eta_1 = -2, \eta_2 = 2, \eta_3 = 2$	Binary variable driving response
Y_1, Y_2, Y_3	$\eta_4 = 2$	Two continuous and ordinal drive response
Treat case 1	$\alpha_0 = -4.9, \alpha_1 = -0.09, \beta_0 = -1.2, \beta_1 = -0.11, \gamma_1 = -0.145, \psi_0 = -0.2, \psi_1 = -0.07$	Odds ratio = 1.217
Treat case 2	$\alpha_0 = -4.9, \alpha_1 = -0.20, \beta_0 = -1.2, \beta_1 = -0.25, \gamma_1 = -0.2, \psi_0 = -0.2, \psi_1 = -0.12$	Odds ratio = 1.426
Treat case 3	$\alpha_0 = -4.9, \alpha_1 = -0.30, \beta_0 = -1.2, \beta_1 = -0.50, \gamma_1 = -0.3, \psi_0 = -0.2, \psi_1 = -0.22$	Odds ratio = 1.794
Treat case 4	$\alpha_0 = -4.9, \alpha_1 = -0.32, \beta_0 = -1.2, \beta_1 = -0.65, \gamma_1 = -0.39, \psi_0 = -0.2, \psi_1 = -0.27$	Odds ratio = 2.007
Treat case 5	$\alpha_0 = -4.9, \alpha_1 = -0.33, \beta_0 = -1.2, \beta_1 = -0.72, \gamma_1 = -0.45, \psi_0 = -0.2, \psi_1 = -0.33$	Odds ratio = 2.198

Table 2. Parameter values for the data generating model in the baseline simulation scenario comparing the performance of the latent variable, augmented binary and standard binary methods for analysing a composite endpoint, where the values correspond to a treatment effect in all components and all components drive response

Purpose	Values
Total sample size	N=300
Intercept	$\alpha_0 = -4.9, \beta_0 = -1.2, \psi_0 = -0.2$
Treatment	$\alpha_1 = -0.28, \beta_1 = -0.35, \gamma_1 = -0.24, \psi_1 = -0.18$
Baseline value	$\alpha_2 = -0.5, \beta_2 = -0.5$
Variance	$\sigma_1 = 4, \sigma_2 = 0.6$
Correlation	$\rho_{12} = 0.5, \rho_{13} = \rho_{24} = 0.35, \rho_{14} = 0.25, \rho_{23} = 0.4, \rho_{34} = 0.3$
Discrete cut-point	$\tau_{13} = -1, \tau_{23} = -0.1, \tau_{33} = 0.45, \tau_{43} = 1.3$
Responder threshold	$\eta_1 = -4, \eta_2 = -0.6, \eta_3 = 0.45, \eta_4 = 0$

Appendix E

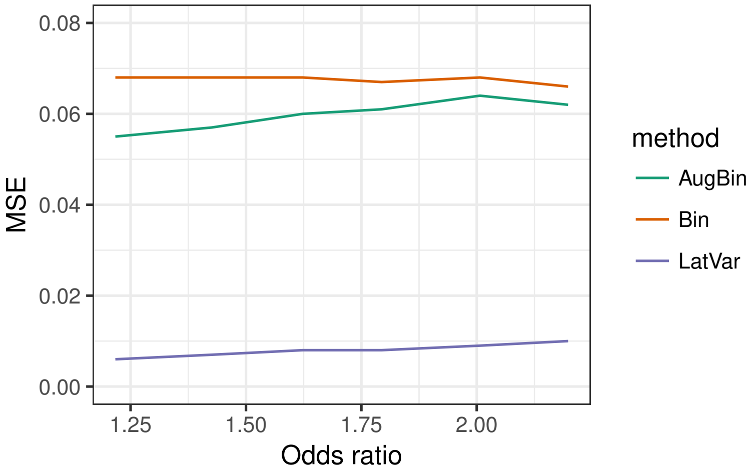

Figure 3. Mean Squared Error (MSE) reported from the latent variable method, augmented binary method and standard binary method for n_{sim}=5000, total sample size N=300 for true odds ratio between 1.2 and 2.2. The composite endpoint of interest contains four components: two continuous, one ordinal, one binary and treatment effects are present in all four components

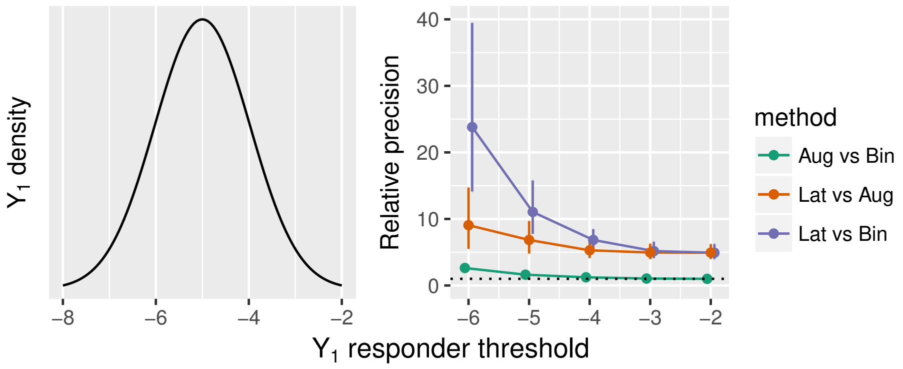

Figure 4. Density of continuous Y_1 variable (left) and estimated relative precision of augmented binary versus standard binary method, latent variable versus augmented binary method and latent variable versus standard binary method as the Y_1 responder threshold η_1 varies between $\eta_1 = -6$ and $\eta_1 = -2$ (right) for n_{sim}=5000 and total sample size N=300. The composite endpoint of interest contains four components: two continuous, one ordinal, one binary and treatment effects are present in all four components

Appendix F

Multivariate skew-normal distribution

To test the robustness of the latent variable method to deviations from joint normality of the components, we can generate the data so that the components are drawn from a multivariate skew-normal. The multivariate skew-normal is an extension of the univariate skew-normal distribution introduced by[3]. They define it as follows. A random vector **Y**=$(Y_1, ...,_k)^T$ has k-variate skew-normal distribution, if its density function is

$$f_k(\mathbf{y}) = 2\phi_k(\mathbf{y}; \Omega)\Phi(\boldsymbol{\alpha}^T\mathbf{y}), \mathbf{y} \in \mathbf{R}^k \tag{F.1}$$

where $\phi_k(\mathbf{y}; \Omega)$ is the probability density function of the k-variate normal distribution with standardised marginals and correlation matrix Ω. The shape parameter $\boldsymbol{\alpha}$ determines the skewness, where $\boldsymbol{\alpha} = \mathbf{0}$ reduces the density in (F.1) to the N($\mathbf{0}, \Omega$) density.

Scenarios of interest are shown in Table 3. The first scenario considers when all four components are skewed. Scenarios 2-3 consider different magnitudes of skew in the latent continuous components only. This tests the robustness of the method to the assumption that the observed discrete variables manifest from continuous variables. Scenario 4 consider skew in the latent components only when the null hypothesis is true. Note that skew has the range (-0.9905,0.9905) so the latter scenario indicates minimal skew.

Results

The bias, coverage, bias-corrected coverage and power are shown in Table 4 for all four scenarios. In scenarios 1-3, the non-normality introduces

Table 3. Simulation scenarios considered to investigate deviations from joint normality for the components of the systemic lupus erythematosus composite endpoint based on the multivariate skew-normal distribution where $\boldsymbol{\alpha}$ determines the magnitude of the skew in each component

Scenario	$\boldsymbol{\alpha}$	Skew	Purpose
skew1	(0.1, 0.1, 0.1, 0.1)	0.083	Skew in all four components
skew2	(0, 0, 0.1, 0.1)	0.026	Skew in discrete components only
skew3	(0, 0, 0.05, 0.05)	0.010	Smaller skew in discrete components only
skew4	(0, 0, 0.05, 0.05)	0.010	Smaller skew in discrete components only in the null case

Table 4. Operating characteristics of the latent variable, augmented binary and binary methods when the components of the systemic lupus erythematosus endpoint are drawn from a multivariate skew-normal, N=300 and $n_{sim} = 1000$

Performance measure	Scenario	Method		
		Latent Variable	Augmented Binary	Binary
Bias	skew1	-0.173 (0.012)	0.041 (0.252)	-0.015 (0.258)
	skew2	-0.103 (0.008)	0.036 (0.251)	-0.020 (0.255)
	skew3	-0.068 (0.008)	0.038 (0.244)	-0.016 (0.245)
	skew4	-0.033 (0.008)	0.007 (0.254)	0.001 (0.255)
Coverage	skew1	0.556 (0.018)	0.933 (0.009)	0.939 (0.009)
	skew2	0.811 (0.013)	0.928 (0.008)	0.941 (0.008)
	skew3	0.884 (0.010)	0.934 (0.008)	0.950 (0.007)
	skew4	0.933 (0.009)	0.923 (0.009)	0.950 (0.008)
Bias-corrected coverage	skew1	0.962 (0.007)	0.929 (0.009)	0.943 (0.008)
	skew2	0.936 (0.008)	0.930 (0.008)	0.943 (0.007)
	skew3	0.940 (0.008)	0.929 (0.008)	0.954 (0.007)
	skew4	0.948 (0.008)	0.926 (0.009)	0.950 (0.008)
Power	skew1	0.897 (0.011)	0.646 (0.017)	0.487 (0.018)
	skew2	0.959 (0.006)	0.637 (0.015)	0.471 (0.016)
	skew3	0.982 (0.004)	0.641 (0.015)	0.495 (0.016)
	skew4	-	-	-

bias which results under-coverage. The bias-corrected coverage is close to nominal for all scenarios however the coverage of the latent variable method is nominal in the null case. This is consistent with our findings when the joint normality assumption is satisfied in that bias is introduced in the estimation of the treatment arm, however the magnitude of this bias is much smaller when the assumptions are satisfied. The augmented binary and standard binary methods behave similarly to when the joint normality assumptions are satisfied, which is expected given that the assumptions of those models are violated in both contexts. The latent variable method still offers large power gains over the other methods.

Table 5 shows the MSE, empirical SE and model SE of the three methods. The latent variable method performs best consistently across these performance measures. The augmented binary and standard binary methods have an MSE across all scenarios of approximately 0.06 whilst

Table 5. Operating characteristics (Monte Carlo standard errors in parentheses) of the latent variable, augmented binary and binary methods when the components of the systemic lupus erythematosus endpoint are drawn from a multivariate skew-normal, N=300 and $n_{sim} = 1000$

Performance measure	Scenario	Method		
		Latent Variable	Augmented Binary	Binary
MSE	skew1	0.039 (0.001)	0.063 (0.003)	0.066 (0.003)
	skew2	0.021 (0.001)	0.063 (0.003)	0.065 (0.003)
	skew3	0.014 (0.001)	0.060 (0.003)	0.060 (0.003)
	skew4	0.010 (0.001)	0.064 (0.004)	0.065 (0.003)
EmpSE	skew1	0.097 (0.003)	0.248 (0.006)	0.257 (0.007)
	skew2	0.102 (0.002)	0.249 (0.006)	0.254 (0.006)
	skew3	0.099 (0.002)	0.241 (0.005)	0.245 (0.006)
	skew4	0.094 (0.002)	0.254 (0.006)	0.255 (0.006)
ModSE	skew1	0.010 (0.006)	0.052 (0.001)	0.064 (0.001)
	skew2	0.010 (0.003)	0.050 (0.001)	0.060 (0.001)
	skew3	0.010 (0.015)	0.048 (0.001)	0.059 (0.001)
	skew4	0.009 (0.004)	0.051 (0.001)	0.063 (0.001)

the MSE of the latent variable method is between 0.01 and 0.04. This indicates that the large reduction in variance is useful despite the introduction of bias. We acknowledge however that this may not hold across all sample sizes ([4]).

Table 6 shows the probability of response in each arm for each of the methods. The findings are consistent with when the assumptions are satisfied. Namely, the latent variable method estimates the probability of response in the control arm well however underestimates the probability of response in the treatment arm. The magnitude of this underestimation is unaffected by the degree of skew or whether the skew is present in the observed continuous components.

The odds ratio treatment effect estimate from each method is shown in Table 7. The latent variable method is biased towards the null, the augmented binary method is biased away from the null. The binary method slightly underestimates the treatment effect in this setting however all are close to true for the null case.
The median relative precision of the methods are shown in Table 8, with the 10th centile and 90th centile values. These are consistent with our

Table 6. Estimated probability of response in the treatment and placebo arms from the latent variable model (Lat Var), augmented binary method (Aug Bin) and standard binary method (Bin) when the components of the systemic lupus erythematosus endpoint are drawn from a multivariate skew-normal, N=300 and $n_{sim} = 1000$

	$Pr(resp \mid T = 0)$				$Pr(resp \mid T = 1)$			
Scenario	True	Lat Var	Aug Bin	Bin	True	Lat Var	Aug Bin	Bin
skew1	0.259	0.263	0.221	0.258	0.365	0.330	0.326	0.359
skew2	0.290	0.287	0.253	0.290	0.398	0.370	0.361	0.392
skew3	0.309	0.302	0.271	0.308	0.418	0.394	0.382	0.413
skew4	0.309	0.299	0.269	0.307	0.309	0.292	0.270	0.307

Table 7. Estimated odds ratio treatment effect from the latent variable model (Lat Var), augmented binary method (Aug Bin) and standard binary method (Bin) when the components of the systemic lupus erythematosus endpoint are drawn from a multivariate skew-normal, N=300 and $n_{sim} = 1000$

	Treatment effect			
Scenario	True	Lat Var	Aug Bin	Bin
skew1	1.640	1.379 (1.140, 1.668)	1.708 (1.093, 2.668)	1.616 (0.985, 2.651)
skew2	1.617	1.459 (1.203, 1.770)	1.676 (1.083, 2.594)	1.586 (0.980, 2.565)
skew3	1.611	1.505 (1.243, 1.822)	1.674 (1.089, 2.572)	1.585 (0.987, 2.548)
skew4	1.000	0.967 (0.807, 1.160)	1.007 (0.647, 1.566)	1.001 (0.613, 1.634)

Table 8. Estimated relative precision from the latent variable model (Lat Var), augmented binary method (Aug Bin) and standard binary method (Bin) when the components of the systemic lupus erythematosus endpoint are drawn from a multivariate skew-normal, N=300 and $n_{sim} = 1000$

	Treatment effect		
Scenario	Lat Var vs Bin	Lat Var vs Aug Bin	Aug Bin vs Bin
skew1	6.903 [5.336, 8.972]	5.579 [4.376, 7.313]	1.231 [1.189, 1.275]
skew2	6.263 [5.013, 7.917]	5.177 [4.096, 6.518]	1.213 [1.178, 1.252]
skew3	6.326 [5.016, 7.995]	5.192 [4.098, 6.548]	1.219 [1.184, 1.257]
skew4	7.384 [5.729, 9.343]	5.985 [4.655, 7.629]	1.231 [1.192, 1.273]

previous findings indicating that the violation of joint normality only affects the bias and not the variance.

Appendix G

We can explore the structure of the data by visualising the 4-D endpoint. Figure 5 shows a plot of the four components in the SLE index. The two panels show taper responders and non-responders, the levels in BILAG are denoted using colours where any coloured data points representing Grade B - Grade E are responders. The response thresholds for the continuous measurements are included, where a patient must be below the threshold to be considered a responder. We can conclude that response is entirely driven by SLEDAI and the taper variable, as there are no PGA non-responders not already accounted for by SLEDAI and no purple data points in the responder quadrant.

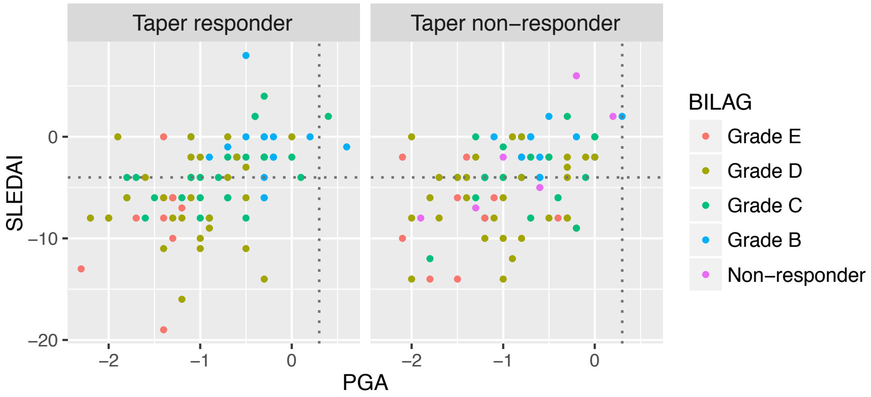

Figure 5. Observed response rates in each of the SLE responder index components in the Phase IIb MUSE trial. SLEDAI is plotted on the y-axis and PGA on the x-axis, along with their corresponding dichotomisation thresholds. Levels of BILAG are represented by different colours and taper responders and non-responders are split across two panels.

Table 9. Observed response rates in each of the SLE responder index components in the anifrolmab 300mg arm and placebo arm of the Phase IIb MUSE trial. SLE index is comprised of a continuous SLEDAI outcome, continuous PGA outcome, ordinal BILAG outcome and binary taper outcome where response in each component is achieved when the patient meets the criteria shown

Components	Response criteria	Treatment arm	
		Anifrolumab	Placebo
SLEDAI	Change in SLEDAI $\leq$ -4	58/89	41/76
PGA	Change in PGA $<$ 0.3	87/89	75/76
BILAG	No Grade A or more than one Grade B	86/89	72/76
Taper	Sustained reduction in oral corticosteroids	53/95	37/87
SLE responder index	Responder in all four components	34/95	18/87

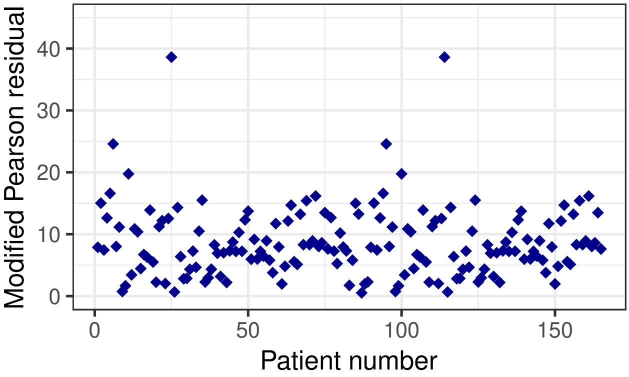

Figure 6. Plot of the modified Pearson residuals from the latent variable model for each patient in the MUSE trial. The residuals highlight that two patients observations are poorly explained by the model but that the model is a good fit for the remaining patients.

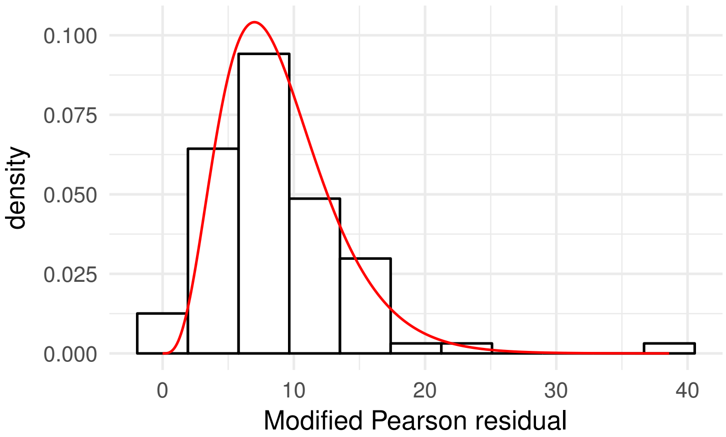

Figure 7. Histogram of the modified Pearson residuals from the latent variable model in the MUSE trial dataset with the corresponding χ^2 density. The modified Pearson residuals follow the distribution of the χ^2 density shown indicating that the model fits well.

References

1. Luijten K, Tekstra J, Bijlsma J et al. The systemic lupus erythematosus responder index (sri); a new sle disease activity assessment. *Autoimmun Rev* 2012; 11(5): 326–329.
2. Samani E and Ganjali M. A multivariate latent variable model for mixed continuous and ordinal responses. *World Appl Sci J* 2008; 3(2): 294–299.
3. Azzalini A and A DV. The multivariate skew-normal distribution. *Biometrika* 1996; 83: 715–726.
4. Morris T, White I and Crowther M. Using simulation studies to evaluate statistical methods. *Stat Med* 2019; 38(11): 2074–2102.
